# Current Nanocarrier Strategies Improve Vitamin B12 Pharmacokinetics, Ameliorate Patients’ Lives, and Reduce Costs

**DOI:** 10.3390/nano11030743

**Published:** 2021-03-16

**Authors:** Marco Fidaleo, Stefano Tacconi, Carolina Sbarigia, Daniele Passeri, Marco Rossi, Ada Maria Tata, Luciana Dini

**Affiliations:** 1 Department of Biology and Biotechnology “C. Darwin”, University of Rome Sapienza, 00185 Rome, Italy; sbarigia.1651648@studenti.uniroma1.it (C.S.); adamaria.tata@uniroma1.it (A.M.T.); luciana.dini@uniroma1.it (L.D.); 2 Research Center for Nanotechnology for Engineering of Sapienza (CNIS), University of Rome Sapienza, 00185 Rome, Italy; daniele.passeri@uniroma1.it (D.P.); marco.rossi@uniroma1.it (M.R.); 3 Department of Biological and Environmental Sciences and Technologies (Di.S.Te.B.A.), University of Salento, 73100 Lecce, Italy; stefano.tacconi@unisalento.it; 4 Department of Basic and Applied Sciences for Engineering, University of Rome Sapienza, 00185 Rome, Italy; 5 Research Center of Neurobiology, University of Rome Sapienza, 00185 Rome, Italy; 6 CNR Nanotec, 73100 Lecce, Italy

**Keywords:** Vitamin B12, cobalamins, nanocarriers, supplementation, malabsorption

## Abstract

Vitamin B12 (VitB12) is a naturally occurring compound produced by microorganisms and an essential nutrient for humans. Several papers highlight the role of VitB12 deficiency in bone and heart health, depression, memory performance, fertility, embryo development, and cancer, while VitB12 treatment is crucial for survival in inborn errors of VitB12 metabolism. VitB12 is administrated through intramuscular injection, thus impacting the patients’ lifestyle, although it is known that oral administration may meet the specific requirement even in the case of malabsorption. Furthermore, the high-dose injection of VitB12 does not ensure a constant dosage, while the oral route allows only 1.2% of the vitamin to be absorbed in human beings. Nanocarriers are promising nanotechnology that can enable therapies to be improved, reducing side effects. Today, nanocarrier strategies applied at VitB12 delivery are at the initial phase and aim to simplify administration, reduce costs, improve pharmacokinetics, and ameliorate the quality of patients’ lives. The safety of nanotechnologies is still under investigation and few treatments involving nanocarriers have been approved, so far. Here, we highlight the role of VitB12 in human metabolism and diseases, and the issues linked to its molecule properties, and discuss how nanocarriers can improve the therapy and supplementation of the vitamin and reduce possible side effects and limits.

## 1. Introduction

Vitamin B12 (VitB12) (also known as cobalamins) is a group of four molecules characterized by a single cobalt atom bound to a corrin-like core, to 5,6-dimethylbenzimidazole (which is bound in turn to a ribose 5-phosphate), and to one variable residue. The latter includes cyanide, hydroxyl group, methyl group, and 5′-deoxyadenosyl group, which determine cyanocobalamin, hydroxocobalamin, methylcobalamin, and 5′-deoxyadenosylcobalamin (also named adenosylcobalamin), respectively. The hydroxocobalamin is the VitB12 found in nature and generally used for administration while cyanocobalamin is an artifact of the extractive process which can be converted after ingestion to the active forms methylcobalamin and adenosylcobalamin [1,2,3].

VitB12 is an essential micronutrient because it is synthesized exclusively by certain bacteria and archaea, and their interaction with animals and plants allows the vitamin to enter the food chain of living beings that need it [4,5]. Furthermore, VitB12 is crucial for life because it takes part as co-factor to two basic reactions of the metabolism: (i) in the form of adenosylcobalamin, it participates in the anabolism of propionate as co-factor for the L-methylmalonyl-CoA mutase which converts L-methylmalonyl-CoA to succinyl-CoA, and (ii) as methylcobalamin, it works as co-factor for the methionine synthase which catalyzes the conversion of homocysteine to methionine. Alteration in the mentioned reactions due to both genetic defect and VitB12 deficiency trigger serious diseases as methionine is required for the synthesis of S-adenosylmethionine (a universal methyl donor involved in a wide range of functions concerning DNA, RNA, proteins, and lipids and hormones), and the conversion of L-methylmalonyl-CoA to succinyl-CoA is essential in fat and protein metabolism. In addition, the absence of VitB12 determines the accumulation of methylmalonic acid and homocysteine that at high levels are toxic [2,6,7]. Both the fact that the organism cannot synthesize the vitamin and the crucial importance in metabolism establish its pivotal role in life.

Although essential, VitB12 absorption results to be a complex and multi-organ process involving the gastrointestinal tract and the alteration of the latter dramatically affects the uptake of the vitamin. For example, the alteration of organ function resulting from ageing causes a vitamin deficiency in the elderly with a prevalence between 1.2% and 40%, depending on they live in community or institutions or are sick or malnourished [6,8,9,10,11]. The deficiency of VitB12 is caused by several diseases such as atrophic gastritis, chronic pancreatitis, inflammatory bowel disease (Crohn’s disease), celiac disease, bacterial infections, or aging. Moreover, gastrointestinal surgery (e.g., stomach reduction or intestinal resection) can lead to VitB12 malabsorption. The reduction of the level of VitB12 may also be due to a dietary deficiency such as in strict vegetarian and vegan diets [6]. Furthermore, aging leads to metabolic and nutritional changes determining an increased vulnerability of vitamin deficiency, and, especially in geriatric patients, these changes can have effects on the nervous system [12]. Frequently, the B-vitamin deficiencies are also caused by a decreased availability associated with an unbalanced diet that can occur in certain subjects (i.e., diabetics, alcoholics, dialysis patients, and patients with gastrointestinal diseases) but may also be caused by the impaired absorption, accelerated usage, and increased hydrolysis, in particular during inflammatory diseases. In addition, the deficit in VitB may be related to the chronic use of several drugs such as omeprazole, methotrexate, colchicine, and proton pump inhibitors [13]. Importantly, the estimated reserves for human beings total 2–5 mg while the daily need is 1–4 µg. Thus, in the case of a deficiency of the vitamin, the synthons can appear after 3–5 years that the malabsorption started [8,10]. 

It is very important to monitor the level of VitB12 in people who could develop a deficiency and, in the case of low levels of VitB12, perform a supplementation. The administration of VitB12 determines a set of problems directly linked to its molecular proprieties that include poor passive gastrointestinal absorption and high excretion. In animal models, it is observed that 98% of administrated vitamin is excreted trough urine [14], while in human beings, it is reported that half-life of VitB12 in plasma is about 6 days and in liver around 12 months [15]. 

Here, we review the malabsorption of VitB12 and its impact on human health. Then, we focus on VitB12 administration and how the use of nanocarriers may ameliorate both the absorption and efficacy of the supplementation.

## 2. Impact of VitB12 and Human Health

The range of effects associated with VitB12 deficiency is very wide and often unrecognized and includes both mild symptoms (weakness, loss of appetite, and weight), and severe ones such as megaloblastic anemia, fertility, embryo development, cancer, and neurological modifications (e.g., numbness and tingling in limbs, depression, confusion, dementia, and optic neuropathy) [6,16,17]. In addition, VitB12 is involved in Peripheral Neuropathic Pain and supplementation can contribute to pain relief [18]. In children, VitB12 deficiency affects thrive and can determine movement disorders and developmental delays. Furthermore, a range of inborn errors that affect VitB12 absorption (Intrinsic Factor deficiency, and Imerslund–Gräsbeck syndrome), transport (transcobalamin deficiency), and intracellular metabolism (combined methylmalonic acidemia and homocystinuria depending on the genes involved) require supplementation/treatment with VitB12 to allow patients to survive [19]. A recent study collected data from preclinical and clinical studies regarding the role of VitB12 in lipid metabolism. Authors highlight that children born from pregnant women that presented VitB12 deficiency have more frequently developed excess fat accumulation and thus a higher risk factor for insulin resistance, type 2 diabetes, and/or cardiovascular disease in adulthood. This evidence strongly suggests an involvement for VitB12 on epigenetic mechanisms although there are very few association studies currently available [20]. As mentioned, in some cases, VitB12 deficiency can be asymptomatic in the onset phase with no obvious symptoms but can cause devastating effects. Here, we delved into the role of VitB12 in the nervous system and fertility since they involve biological barrier structures that need to be overcome and can well show the difficulties for specific treatment with VitB12.

### 2.1. VitB12 Affects Pregnancy, Fertility, and Embryo Development

Deficiency in VitB12 is common in pregnant women and is associated with high homocysteine levels (hyperhomocysteinemia), which is a risk factor for recurrent spontaneous abortion (RSA) [21,22]. Molecular defects involved in VitB12 transport and/or uptake can cause cellular deficiency (see next paragraph for the processes involved in absorption). For example, it has been observed that polymorphisms in the protein transcobalamin II (a carrier of VitB12 in the blood flow) and transcobalamin receptor TCblR (which binds the complex VitB12/transcobalamin II allowing the receptor-mediated endocytosis in target organs) are associated with RSA [21]. Moreover, altered levels of VitB12 are correlated with metabolic insulin resistance, gestational diabetes mellitus [23], and preeclampsia [24].

Substantial evidence has demonstrated that diet in pregnancy has relevant effects on the development of the child. In particular, the VitB12 levels are relevant for axon myelination considering their role in the metabolism of both fatty acids and amino acids. Consequently, it is likely to be particularly important in the growth and development of the fetal brain as suggested by the impaired cognitive function in childhood such as speech and mathematical abilities as a consequent of VitB12 deficiency in the mother during pregnancy [25,26,27].

Albeit that a review of the health effects of VitB12 by the World Health Organization concluded that additional research into the possible adverse effects of deficiency during pregnancy is needed [28], several studies have shown evidence of adverse effects identifiable at birth for children born by women who were deficient in VitB12, including preterm delivery [29] and neural tube defects [30].

Furthermore, VitB12 is also fundamental for sperm function, and hyperhomocysteinemia is toxic for sperm [31]. Consequent hyperhomocysteinemia, due to VitB12 deficiency, affects the nitric oxide synthase leading to decreased nitric oxide levels. In turn, the resulting enhanced level of oxidative stress determine oxidative damage to sperm and triggers systemic inflammation causing altered sperm motility. Therefore, VitB12 deficiency in general negatively affects spermatogenesis, causing atrophy of seminiferous tubules and aplasia of spermatozoa and spermatids [32,33].

Altogether, these data suggest that the level of VitB12 may have a relevant impact on fertility, pregnancy, and correct embryo development.

### 2.2. VitB12 Alterations and Nervous System Pathologies

Vitamin B complex is essential for the correct functioning of the nervous system, and its deficiency has recently been recognized as risk factors for stroke, dementia, and peripheral neuropathy (PN) [34]. VitB12 has been strictly associated with cognitive functions and its low levels represent a risk factor for Alzheimer’s disease (AD). Albeit that a lot of studies suggest that VitB12 supplementation fails to fully protect from AD, its role in the prevention and treatment of cognitive impairment should not be underestimated [35,36]. The importance of B vitamins in the context of nerve function is also described for other neurological diseases, such as Wernicke’s encephalopathy, depression, subacute combined degeneration of the spinal cord, and, as mentioned, PN [34].

The relevance of the VitB12 as a modulator of neurospecific functions is due to its role of co-factor in the conversion of L-methylmalonyl-CoA to succinyl-CoA and homocysteine to methionine. Indeed, the association between the dysfunction of the methionine–homocysteine cycle, cognitive impairment, and low levels of VitB12 protein has been reported [37,38]. Furthermore, VitB12 deficiency determines the accumulation of methylmalonic acid, which is toxic in particular for myelin, hence the issues regarding the optic and peripheral neuropathies [39]. Mechanisms involved in the central nervous system (CNS) impairments due to deficiency of VitB12 are not completely described, yet. It is known that a scarce availability of VitB12 leads to a decrease of S-adenosylmethionine and in turn hypomethylation and demyelination of CNS and a reduction in the synthesis of phospholipids [40]. Researchers have highlighted that VitB12 is essential for Schwann cells and oligodendrocytes (cells producing myelin), thus having a crucial role in myelin formation and remyelination and regeneration of nerves after peripheral injury [41,42,43]. Deficiency of VitB12, besides causing demyelination, also leads to incorporation of abnormal fatty acids into neuronal cells and modify the level of some neurotransmitters [41,42,43]. Moreover, the unavailability of VitB12 determines an accumulation of homocysteine which can affect the oxidative stress and modify calcium influx (through stimulation of N-methyl-D-aspartate receptor) [40]. Altogether, these events lead to neurodegeneration and might cause cerebral dysfunction, brain atrophy, and dementia frequently observed during the decrease of vitamin [40,41,42]. The use of B vitamins, especially vitamins B1 (thiamine), B6 (pyridoxine), and VitB12, for the treatment of nervous system pathologies has been proposed. However, several reports indicate that the specific supplementation of these vitamins can interact synergistically improving neuropathy, motor control, nociceptive, and neuropathic pain. In addition, high doses or long-term treatment may have toxic effects. For example, VitB12 administration can be associated with dermal adverse effects (e.g., acne). The risk appears to be higher with hydroxocobalamin than cyanocobalamin [44].

Considering these aspects, more precise methods of VitB12 administration that may increase absorption, prolong its effects (also in the case of a low dose) and improve its specific deliver to target organs is needed.

## 3. A Complex Route for Absorbing VitB12

**Figure 1:**
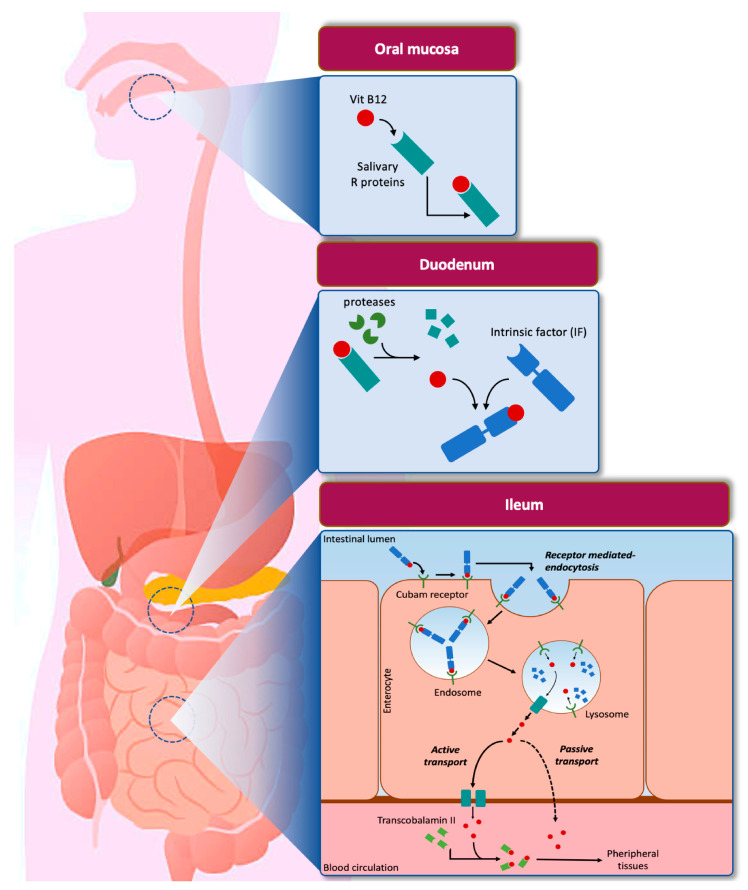
VitB12 uptake pathway. The scheme summarizes the main stages of VitB12 absorption and describes the factors and organs involved. See text for the complete description.

In human beings, a finely regulated multi-step process determines VitB12 uptake and an alteration in the functioning of mechanisms involved in absorption determines a deficiency (Figure 1). Although a very small amount of VitB12 can be absorbed by a passive diffusion process (~1.2%), its major uptake occurs through a specific intestinal mechanism [45]. After food ingestion, VitB12 associates to the R-protein (transcobalamin I), a carrier secreted by the oral mucosa with a glycosylated structure, resistant at low pH, that protects the vitamin from the acid environment of the stomach [46]. Then, the formed complex moves forward to the intestine. In the duodenum, R-protein is degraded by pancreatic proteases and the released VitB12 associates promptly to the glycoprotein Intrinsic Factor (IF). In the ileum, the VitB12/IF complex is recognized by the cubam receptor of enterocytes and absorbed through receptor-mediated endocytosis. In particular, the cubam receptor is composed of a transmembrane protein (amnionless protein, AMN), which drives internalization, and an outer protein, cubilin, which is responsible for binding the VitB12/IF complex. In lysosomes, IF is degraded and VitB12 is actively transported into the cytoplasm by a trans-membrane protein encoded by *LMBRD1* gene [47]. Once in the cytoplasm, free VitB12 is conveyed through the basolateral site of the enterocyte by active transport (mediated by a multispecific membrane transporter, multidrug resistant protein 1, MRP1) or passive transport to the blood flow. In the blood flow, the VitB12 can bind the carriers transcobalamin II (TCII) and haptocorrin (HC) with a different degree of affinity. In particular, TCII, secreted by vascular endothelial cells, binds the vitamin B12 with high affinity to form a complex, holotranscobalamin II (HTCII), which is the bioavailable circulating form of VitB12, and it is directed towards the peripherical cells to be absorbed through a receptor-mediated endocytosis process. The remaining VitB12 binds with low selectivity to HC, perhaps to remove damaged vitamin molecules or to act as a reservoir. However, the role of HC is still poorly understood (reviewed by [48]). 

VitB12 is a water-soluble vitamin, and its excess is generally excreted through urine. However, thanks to a particular protein called megalin, located in the proximal tubule of the kidney, reuptake occurs. Megalin has a high affinity for HTCII and allows the recovery of the active form of VitB12 in the bloodstream or within the cells [41]. Oral absorption of VitB12 starts from the mouth, passes through the gastrointestinal tract, reaches the bloodstream, and then the vitamin is targeted to the specific organs. This multi-step mechanism requires different interactions with specific molecules and a single-stage invalidation determines an unaccomplished process. For example, IF unavailability represents the main cause of VitB12 deficiency. Intramuscular injection of VitB12 allows bypassing several phases involved in oral absorption; thus, for this reason, it has been preferred rather than oral prescription [9,11,49]. 

## 4. Current Medical Practices

Some studies collect data regarding the comparison of intramuscular and oral administration of VitB12 but they result very heterogenic to make a meta-analysis [9,11]. For example, in some reports, healthy individuals or patients were administrated with cyanocobalamin and in other ones with hydroxocobalamin. These two forms of VitB12 can persist differently in the body: in particular, hydroxocobalamin is retained longer, allowing a 3-month interval between a dose and the following one [50]. Furthermore, the studies involved a small number of participants and the follow-up period is limited [9,11]. For example, Kuzminsk and co-workers compared daily oral administration (2000 µg) of cyanocobalamin with monthly intramuscular injection (1000 µg) in recently diagnosed cobalamin deficient patients [51]. Bolaman and colleagues treated megaloblastic anemia patients with oral and intramuscular progressively-reduced-frequency administration of cyanocobalamin (1000 µg) [52] for 90 days. In Saraswathy’s research, the scientists evaluated daily oral prescription (1000 µg) vs. weekly intramuscular injection (1000 µg) [53]. Castelli’s paper reports data from healthy people with low cobalamin administrated who received oral VitB12 (1000 µg/day) or intramuscular VitB12 (1000 µg every 10 days) [54]. All mentioned studies conclude that oral administration is as effective as an intramuscular injection even if the oral dose used in the studies considerably differed thus making estimation of the appropriate treatment difficult. Although a dose-finding trial established that about 600 µg of cyanocobalamin improves of 80% plasma parameters associated with a VitB12 deficiency [55], some medical association suggest 1000 μg/day in the case of pernicious anemia or general malabsorption [56]. Results recently published from the Project OB12 (Oral/Intramuscular B12 to Treat Cobalamin Deficiency), a clinical trial with a follow-up of one year and involving 283 participants deficient for VitB12 and being ≥ 65 years of age, showed that oral administration and intramuscular injection do not give different outcomes at 8 weeks of treatment while at 52 weeks oral administration showed a difference by 10% less of VitB12 serum level that was still sufficient to accomplish the vitamin deficiency. Moreover, the study reported a higher preference of participates (30%) to receive oral prescription [57]. Finally, it must be stressed that Kuzminsk and co-workers observed that at 4 months of treatment, the oral prescription gives 3 times the serum VitB12 value compared to the parental administration although the latter is in the normal range level [51].

Intramuscular injection is the first-line treatment for VitB12 deficiency, but it is very stressful for patients (especially for children that need long-life treatments) and expensive. Oral administration, which could ameliorate the patient’s life and save money, is not preferred although it is well known that VitB12 can be passively absorbed even in patients affected by pernicious anemia or after gastroduodenal surgical resection [11]. The choice of the intramuscular option may infer from both a lack of clear studies as well as knowledge regarding the gastrointestinal pathologies which can produce a high degree of enzymatic degradation and chemical instability by changing the pH of the environment which can interfere with the uptake [58,59]. To overcome these issues and concerns, recently, intranasal and sublingual VitB12 administrations are being developed and are emerging as being very effective due to their stressless nature. The comparison between intranasal and intramuscular prescription lacks big studies and the available data suggest that intranasal administration has a low bioavailability (between 2% and 5%) which is an appropriate quantity to fulfil the deficiency [60,61]. We must add that in Tillemans’s study, they used hydroxocobalamin solution for injection and cyanocobalamine for spray; these VitB12 forms persist differently in the body and can underestimate the intranasal administration [50,61]. Interestingly, two small studies carried out on children with VitB12 deficiency or adults with ileum resection showed that intranasal treatment with hydroxocobalamin increases the plasma level of the vitamin proportionally to the dose [62,63]. Although a direct comparison between the reports is very difficult, the patients evaluated in Estourgie-van Burk’s study had a very heterogeneous treatment but the average dose of hydroxocobalamin in the first week of therapy was 12 mg per week, while Slot and co-workers administrated 1.5 mg per week; the results collected showed a vitamin plasma level of 2000 pmol/L and 250 pmol/L, respectively [62,63].

A very recent study reports the comparison of sublingual vs. intramuscular administration of cyanocobalamin for the treatment of patients with VitB12 deficiency showing that both treatments have similar outcomes [64]. Moreover, it is observable as a dose/response effect in sublingual prescription [65]. 

Current medical practices refer to protocols developed in the past which, although completely functioning, do not consider the quality of patients’ life, the cost of the treatment and, although high doses of VitB12 are considered safe, only a few papers report pharmacokinetics/bioavailability of the drug.

Several efforts have been done to improve VitB12 delivery and new strategies include the use of nanocarriers.

## 5. Nanotechnologies and VitB12

Nanocarriers in the pharmaceutical application have been developed to avoid nonspecific distribution and uncontrolled release of drugs that occurs in conventional administration. Targeted delivery of VitB12 is a major focus of modern prescriptions: for instance, conveying the vitamin to the bone marrow and nerve cells is currently a pharmacological challenge for myelin recovery [66].

Nanocarriers are organic (e.g., liposomes, micelles, hydrogels and polymer, dendrimer, and solid lipid nanoparticles) or inorganic (metallic, magnetic, and semiconductor nanoparticles, such as carbon nanotubes) nanoparticles, typically 1–100 nm in size, which, under appropriate modifications, could be considered as smart drug delivery systems [67]. Compared with traditional drug delivery systems, the use of nanocarriers displays many advantages, e.g., a prolonged drug half-life, controlled release and enhanced drug absorption, improved drug stability, limited drug side effects, and targeted delivery [68]. For this reason, nanocarrier-based drug delivery systems may represent a novel strategy for the treatment of several human diseases, including tumors, diabetes, and others [69]. Not all types of nanocarriers are reliable as drugs carriers; indeed, an ideal nanocarrier as a drug delivery system needs to be modified or functionalized and should fulfill specific criteria. First, they must avoid biological barriers and immune system responses. For example, PEGylation is a modification strategy that allows nanocarriers to escape cleansing processes, such as the reticuloendothelial system; however, it reduces the drug uptake by cells [70]. Second, nanocarriers should be accumulated specifically at the targeted site: this could be possible by modifying nanocarriers with ligands matching overexpressed receptor proteins, in order to recognize specific target cells. Last, nanocarriers must release the cargo at the targeted site at the right concentration under several different stimuli (e.g., redox, temperature, light, electric field, and others). To make nanocarriers responsive to those stimuli, various chemical groups can be grafted on their surface [71]. 

Although the prospect of nanocarrier-based delivery systems is quite promising, their toxicity and biocompatibility in human organs is a major concern: nanoparticles are absorbed, released, and metabolized in vivo, but these processes are still largely unknown [67]. Generally, the absorption process of nanocarriers is complex and depends on multiple factors, including the route of administration. By oral administration, nanocarriers can be absorbed into the blood through several pathways, like chemical and enzymatic barriers in the gastrointestinal tract, via passive cross-cell diffusion, paracellular pathway transport, carrier-mediated transcytosis, or M cell absorption. The latter allows nanocarriers to directly enter the lymphatic system, avoiding the first-pass effect and improving the bioavailability of the drug [72]. Nanocarriers accumulate in different vital organs, such as the lungs, heart, kidneys, spleen, and liver, and their deposition is the main cause of their potential toxicity. As a matter of fact, their safety and their impact on the body are still under investigation, and many in vitro and in vivo studies have been performed, despite studies in the human body are limited [73]. All the engineered nanocarriers exhibit some degree of toxicity, which is predominantly related to their size (the most important parameter in toxicity assessment of nanocarriers), shape, and surface charge, but also the route of administration (e.g., intravenous administration brings more medical complications compared to oral administration) and the dose of drugs [74]. Although efficacious, safety remains a primary requirement for biomedical application to be considered. However, by virtue of their precise targeting and controlled release, nanocarriers could represent a promising alternative approach to conventional therapies for the treatment of certain diseases, and more extensive research is needed in order to understand the pharmacology and toxicology of nanoparticle formulations and improve their effectiveness and safety in vivo.

**Figure 2:**
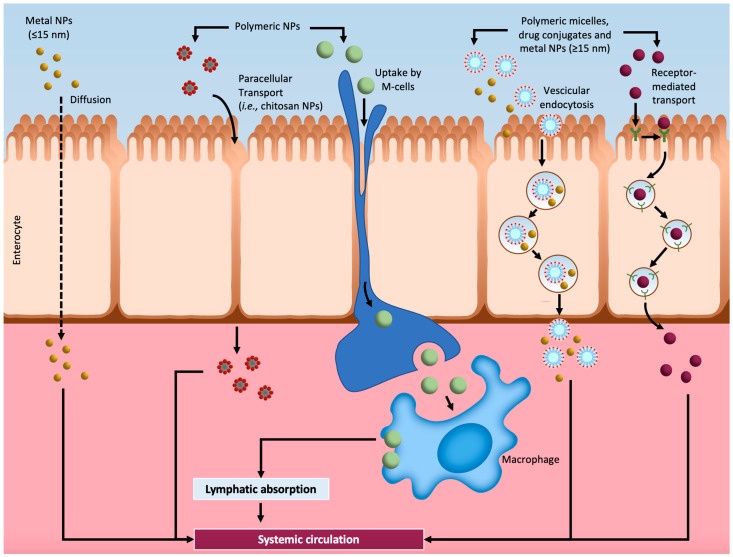
Nanocarriers uptake. The picture reports the general uptake of nanocarriers in the gastrointestinal tract (which is the main target for several nanoparticles that involves oral administration) (adapted from the works in [75,76,78,84]).

The design of the “perfect” nanocarrier should consider several aspects of the possible biological interactions. For example, nanoparticles that are supposed to be absorbed in the small intestinal tract after oral administration pass through the mouth and stomach, arriving in the small intestine. These organs are characterized by specific environments that dramatically differ. Just to mention the pH, in the mouth it is 5–8, in the stomach 1–3, while in the small intestine 6.3–9. Furthermore, the composition of the lytic enzymes responsible for the digestion of food is very diverse. pH and lytic enzymes are just a part of the complex picture. Indeed, researchers still do not completely understand, and cannot characterize, how specific nanocarriers are absorbed or how they interact with tissues [75]. In Figure 2, we report some mechanisms that allow nanoparticles to go beyond the intestinal barrier. Nanoparticles that are very small can diffuse throughout the epithelial barrier, while others can interact with tight junction protein thus translocating through intercellular space. In some cases, nanoparticles are uptake through Microfold cells (or M cells) (such as CaCO_3_) or enterocytes can internalize them by the vesicular endocytosis process. Furthermore, when a nanoparticle is associated with a drug specifically targeted for a receptor, its uptake may involve a receptor-mediated endocytosis process [75,76,77,78]. Finally, note the role of the P-glycoprotein (Pgp). It is an ATP-dependent transporter in the apical surface of enterocytes and is responsible for the efflux of xenobiotics from the cell to the intestinal lumen. To elude Pgp, different strategies have been used including the co-administration of Pgp inhibitors [79].

Thiolated polyacrylic acid particles (PPA) have been used to formulate an oral prescription that absorbed at the gastrointestinal level. PPA particles display mucoadhesive features, permeation-enhancing and efflux pump inhibition capacity (which improves absorption and retainment of the drug by the cell), and controlled-release properties. VitB12 loaded in PPA–cysteine conjugates exhibits both an improved permeation ability in Caco-2 cell and rat intestinal mucosa models [80] and a 3-fold increased bioavailability in an in vivo rat model [81]. The authors used particles with dimension ranging from nanoscale to microscale but PPA-cysteine can be synthesized in a smaller size of 200 nm [82]. 

Another strategy for VitB12 delivery involves nanoengineered polymeric capsules (NPCs). These are made up of overlapping polymeric shells which form a hollow container that can be filled with a desirable substance. Maiorova and co-workers produced nanocapsules of 50–300 nm diameter in the form of nanoaggregates embedded with VitB12. The latter are obtained using colloidal CaCO_3_ as templates for the shell formation, containing alternate layers of poly(allylamine hydrochloride) (conjugated with tetra-methylrhodamine isothiocyanate) and poly(sodium 4-styrene sulfonate). Obtained particles were left in a VitB12 solution to accomplish the load of vitamin [66]. 

Similar to NPCs, lipid matrixes have also been used to encapsulate VitB12. The highly organized internal structure allows the drug to be slowly released. Two examples are phosphatidylcholine/cholesterol/phosphatidylglycerol liposomes [83] and cubosomes [66]. The latter are self-assembled structures formed by amphiphilic phytantriol molecules in water excess which have a high interfacial surface that increases the ability to encapsulate a drug compared with liposomes [66]. Interestingly, these carriers can be used to target a drug both exploiting the features of a specific disease or acting external with physical action. For example, magnetic nanoparticles can be incorporated in the capsules and, thanks to an external magnetic field, they can be conveyed in the site of interest. Furthermore, the nanocapsule release is a consequence of the formation of pores on their surface that can be induced by changing several factors. For instance, the decrease in pH determines permeability of the nanocapsules. Thus, diseases characterized by a reduction of pH can determine a natural release of encapsulated substances; again, in arthritis, metalloproteins display higher activity, and covering the nanocapsule with collagen, which is degraded by metalloproteins, will ensure a release of the drug [66]. 

Nanoclays have been investigated as potential carriers for VitB12 [85]. Nanoclays are nanoparticles of layered mineral silicates with a thickness of about 1 nm and surfaces between 50 and 150 nm [86]. Nanoclays derived from montmorillonite were used as an adsorbent for VitB12. The aim of this study was to investigate the adsorption mechanism and arrangement of VitB12 onto/into montmorillonite. Montmorillonite has been chosen because it does not interfere with intestine functions during digestion and does not have side effects and for its interesting properties related to the high surface area and ion-exchange capability [85]. The external surfaces of the nanoclay determine hydrophobic interaction while at the edges or interlayer spaces it forms cationic bridges; the absorption of VitB12 in the nanoclay can be modulated changing the mentioned sites, thus controlling its properties as nanocarrier in order to produce nanoparticles for improving bioavailable and controlled release of VitB12, or the drug of interest, for oral administration [85]. 

A thermo-responsive polymer made up of poly (N-isopropylacrylamide) (PNIPAM) and poly (ethylene oxide) (PEO) has been used to optimize the release of VitB12. The PNIPAM/PEO scaffold shows a slower release of the drug at 37 °C than 25 °C, suggesting that this nanostructure is suitable as a carrier for drug delivery in humans. Furthermore, when PEO is used as a matrix to facilitate the formation of PNIPAM, it affects the diameter of the fibers and the latter can attribute different features to the carrier. In particular, VitB12 embedment in PNIPAM/PEO shows different release kinetics: the increase of the amount of PEO (ranging from 1% to 4%), and the concomitant impoverishment of PNIPAM (ranging from 10% to 4%), reduces the diameter of the fibers (the smaller diameter per groups ranges from 250 nm to 100 nm) and modify the in vitro release of VitB12 (with the smaller fibers having the higher cumulative release). Furthermore, the VitB12 loading amount can affect the release: the higher amount loaded of vitamin determines the lower cumulative release [87].

VitB12 has anticancer effects, but one main issue is that cells do not uptake the needed amount of vitamin to exert an antitumor effect when it is administered as such. Genç and co-workers showed as VitB12-loaded solid lipid nanoparticles (VitB12-SLNs) can improve the vitamin uptake by cells and the anticancer properties of the vitamin and be specific for cancer cells [88]. They performed some in vitro experiments using transformed-cells 5RP7 (H-ras transformed-rat embryonic fibroblast) and NIH/3T3 (mouse embryonic fibroblast) as counterpart and treated both cells with VitB12 and VitB12-SLNs. The VitB12-SLNs has an average diameter of about 650 nm with a shorter dimension of about 100 nm (this dimension has been inferred by the pictures in the paper). The release of VitB12 from the complex is pH-dependent: in PBS solution, after 90 min, at pH 7.4, it is observable a release of 50% while in the same condition but at pH 6.8 the quantity emitted is more than 80%. Only the VitB12-SLNs complex is able to enter the cell by passive diffusion and accumulate in the cytoplasm (in both cell lines) while when VitB12 is administrated as such, it is found around the cell in the extracellular space. H-ras 5RP7 cells formed phagocytic vacuoles and lamellar body and underwent apoptosis while NIH/3T3 were not affected (even if, at high dosage, an increased number of mitochondria and deformation mitochondrial membrane in NIH/3T3 cells was observed) [88]. The authors do not explain the cause of the difference in toxicity for the two cell lines, but it is our opinion that the more acidic pH of cancer cells respect normal ones could increase the release of VitB12 from the complex and exert the anticancer effect. Although this approach was designed for the treatment of cancer cells, the results show that normal cells can internalize the complex as well, and thus it could give advantages in the treatment of congenital diseases concerning the transport of VitB12.

**Figure 3:**
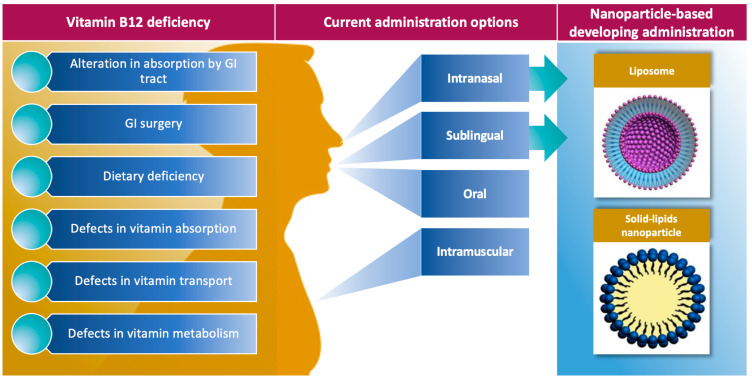
Current application of nanocarriers in VitB12 administration. The scheme summarizes the causes of VitB12 deficiency, the current administration options, and the nanoparticle-based developing administration.

The present options of VitB12 administration consider sublingual, intranasal, and oral administration besides intramuscular injection (Figure 3).

Nanocarriers currently available and tested on human beings have been developed avoiding uptake issues deriving from impairment of the gastrointestinal tract and thus bypass it by exploiting the oral–buccal mucosa accessing. Indeed, lipid-based nanoparticles have been used to deliver lipophilic active substance via the facial lymphatics and in turn into the systemic circulation [58,89]. In a recent study, the authors have compared different oral preparations including nanocarriers to administer VitB12 in healthy people [90]. In particular, they observed the level of VitB12 in the plasma after six hours of the administration of 1000 mg of VitB12 given in four different buccal preparations including two oral spray formulations consisting of methylcobalamin embedded in liposomes (approximately 100 nm) and methylcobalamin encompassed in 20 nm nanoparticles (NanoCelle) made up of a hydrophobic core and an outer hydrophilic shell [90]. Both mentioned formulations are absorbed through the buccal mucosa. The comparison of VitB12 level in plasma showed that at 6 h of administration of the 1000 mg of vitamin, the engineered administrations were performing well. Indeed, NanoCelle and liposome formulation had a higher vitamin uptake (about 28% and 14% respect the baseline, respectively) while emulsion formulation, which allows VitB12 to passively diffuse through the sublingual mucosa, showed a 10% increase. Finally, tablet administration (absorbed through the gastrointestinal tract) had the lowest increase in vitamin plasma content (5% respect baseline). Interestingly, the administration of 5000 mg of vitamin (high dosage) through a dissolvable (chewable) tablet which allows sublingual mucosa passive diffusion showed a comparable level to the nano-formulations. Altogether, it suggests that oral–buccal mucosa can ensure a higher uptake of VitB12 respect gastrointestinal tract and that engineered nutraceutical can be used at lower dose respect conventional preparation to obtain the same results [90].

Furthermore, we mention nanocarriers produced for delivering several substances that have been projected by using the VitB12 as an enhancer of their proprieties. For example, layer-by-layer coated calcium phosphate nanoparticles functionalized by grafting VitB12 have been developed to allow insulin to be orally delivered. The conjugated vitamin permit to exploit of the receptor-mediated endocytosis in epithelial cells and improve insulin absorption through nanoparticles capsules of about 4.3-fold, thus representing a promising vehicle for insulin or other peptide delivery [91]. With the same aim, also VitB12-coated nanospheres have been produced [92]. Chalasani and co-workers produced dextran nanoparticles subsequently modified to provide carboxylate groups for linkage to amino VitB12 derivates [92]. Equally, solid lipid nanoparticles coated with VitB12-stearic acid conjugate have been produced to encapsulate amphotericin B to improve the low oral dose treatment against visceral leishmaniasis [93]. VitB12, besides improving absorption through the exploitation of the receptor-mediated uptake, is also used to enhance the solubility of components of nanoparticles [94].

### How to Monitor the Deficiency of VitB12: Involvement of Nanotechnologies

In addition to nanocarrier-mediated delivery, different nanotechnology-based solutions have been proposed for the detection of VitB12 in foods and food supplements as well as in human tissues. For example, these techniques involve the use of nanoparticles used in fluorescence-based methods, metal nanoparticles and nanostructured surfaces enhancing the sensitivity of spectroscopic methods through surface plasmon resonance (SPR), nanostructured surfaces for the realization of electrochemical sensors, nanoparticle assisting analytical methods like solid-phase extraction and high-pressure liquid chromatography (HPLC) analysis.

Zhang et al. realized phosphorus and nitrogen dual-doped carbon quantum dots (QDs) with size doing range between 2 and 6 nn and mean diameter of 3.4 ± 0.2 nm, as determined using transmission electron microscopy (TEM) and atomic force microscopy (AFM). These doped carbon QDs were demonstrated to behave as effective fluorescent nanoprobes for the sensitive and selective detection and quantification of VitB12 in drug samples and human cells such as hepatocarcinoma (type SMMC7721), pulmonary epithelial (type BEAS-2B), adenocarcinoma (type A549), and pheochromocytoma (type PC12) cells, with a limit of detection (LoD) of 3.0 nM [95]. Long et al. realized silicon QDs with a size of 4–5 nm which they employed to detect VitB12, the presence of which was demonstrated to quench the fluorescence of silicon QDs due to the inner filter effect. Fluorescence quenching efficiency was observed to monotonically increase as a function of the VitB12 concentration, thus allowing the detection of VitB12 with an LoD of 158 nmol L^−1^ [96]. Pourreza et al. demonstrated a fluorescence method based on the use of chitosan-embedded Ag nanoparticles which interact with VitB12, cloud point extraction, and the subsequent detection using fluorescence. The method was demonstrated to allow the selective determination of VitB12 in human plasma and urine with an LoD of 0.035 ng mL^−1^ and a limit of quantification (LoQ) of 0.120 ng mL^−1^ [97]. Nanosized materials are characterized by a high surface-to-volume ratio, which increases as the dimension of the nanoparticles decreases. Therefore, the deposition of nanoparticles on electrodes can result in the production of nanostructured electrochemical sensors with enhanced active surface. This approach has also been demonstrated for VitB12 electrochemical detection. For example, Akshaya et al. have recently reported the realization of electrochemical sensors obtained by electrodeposition of palladium and gold nanoparticles on polypyrrole-modified carbon fiber paper electrodes, which they employed to detect methylmalonic acid, a marker revealing VitB12 deficiency. The method was demonstrated to allow the determination of methylmalonic acid in human blood serum and urine with an LoD of 1.32 pM. Due to their tree-like structure, dendrimers represent valid solutions to increase the active surface of electrochemical sensors [98]. Parvin et al. fabricated ferromagnetic nanoparticle-incorporated triazine dendrimer and polypyrrole on gold electrodes to realize an electrochemical sensor which was demonstrated to allow the selective detection of VitB12 with LoD of 0.62 nM [99].

Optical spectroscopy techniques, including chemiluminescence, absorption, fluorescence, and Raman spectroscopy, have been demonstrated to be powerful methods for the detection and quantification of VitB12 in food matrices and human tissues such as blood, serum, and urine [100]. Nanotechnologies offer unique solutions to improve the sensitivity of these methods by taking advantage of the enhancement of the electromagnetic signal in presence of metal nanoparticles or surfaces with nanosized features due to the surface plasmon resonance (SPR) effect. For example, Radu et al. produced surface-enhanced Raman spectroscopy (SERS)-active substrates realizing 2D gratings with a period of 250 nm and depth of 100 nm using e-beam lithography, vacuum evaporation, and ion-beam etching on fused silica substrates eventually covered with a 40 nm thick Ag layer [101]. These nanostructured substrates were used to detect VitB12 in food matrices, e.g., fortified cereals, starch, and sugar, with a LoD of 70 nM. Furthermore, an SPR method has been proposed for the detection of different vitamins B, including B2, B9, and B12 in infant formula and milk samples, which was demonstrated to maintain its performances in terms of specificity and selectivity over several months, with a LoD of 0.25 pg mL^−1^ for VitB12 [102]. The use of enhanced Raman spectroscopy to quantify VitB12 in multivitamin complexes was also successfully demonstrated by Ibáñez et al., who realized screen-printed Au electrodes to be used in the electrochemical SERS detection of VitB12 with an LoD of 4–7 nM [103]. Wiesholler et al. realized active substrates depositing Tm^3+^-doped NaYF_4_ nanoparticles on arrays of Au nanotriangles on glass substrates, which were used to detect VitB12 in serum analyzing the near-infrared (NIR) to ultraviolet (UV) luminescence upconversion, obtaining an LoD of 3.0 nmol L^−1^ [104]. 

An integrated nanotechnological approach was used by Kamruzzaman et al. who realized a microfluidic device containing luminol and AgNO_3_, where VitB12 was detected due to the chemiluminescence reaction in which luminol was oxidized by Ag^+^ ions in presence of colloidal Au nanoparticles with sized of 11 ± 1 nm with catalytic function. VitB12 was detected in VitB12 only and multivitamin tablets with an LoD of 0.04 ng mL^−1^ [105].

Finally, an application of nanotechnology in the field of VitB12 detection is represented by the development of analytical methods which involve the use of nanomaterials and nanosystems to obtain improved performances. An example is represented by the use of magnetic nanoparticles to assist solid-phase extraction in order to detect VitB12 at trace levels using HPLC. For example, Ulusoy et al. reported the realization of core–shell nanoparticles constituted of a magnetic core of Fe_3_O_4_ and a nanocarbon-based shell formed by a layer of multiwalled carbon nanotubes (MWCNTs) and a layer of nanodiamond (ND). These nanoparticles were used in the magnetic solid phase extraction prior to HPLC analysis of VitB12 in different food matrices, e.g., infant formula and breakfast cereals with wheat, as well as certified reference materials, demonstrating an LoD of 2.85 ng mL^−1^ for the proposed method [106].

## 6. Overcoming the Blood–Brain Barrier (BBB): A Pharmacological Challenge for VitB12 Treatment Solvable by Nanotechnology

BBB and blood-testis barrier are biological structures that protect related organs and establish actually a major biological structure that imposes a major barrier to drug delivery [107]. As previously discussed, deficiency of VitB12 leads to defects in several organs and, in particular, to specifically target treatments to the brain or the testis sites is currently a pharmacological challenge.

Neurological damage following the lack of vitamin revels its strong involvement in the physiological activity/balance of the CNS more finely than in other tissues. Despite the well-characterized mechanism regarding the intestinal uptake of the vitamin, its transported by other organs is unclear and, in particular, how VitB12 overcomes the BBB and reaches the CNS remains unknown [41], and this makes a limit for targeted administration of the vitamin to the CNS which could lead to a high benefit. Between noninvasive treatments, nanoparticles seem to be a promising approach. The very small NP get an advantage from their size: for example, gold NPs that have a 10 to 20 nm diameter, effectively cross the BBB. On the contrary, larger particles around 50 and 100 nm diameter have difficulty reaching the brain [108]. It must be underscored that the shape of NPs can also affect cellular internalization pathways [109]. A very recent and innovative approach uses naturally occurring cell-derived membrane vesicles (CMV). A particular class of CMVs are exosomes, i.e., nanosized vesicles deriving from endocytotic compartments. CMVs are actively secreted by cells and can transport endogenous molecules and macromolecules such as small metabolites and proteins and nucleic acids between cells both intra-organs and inter-organs. The contribution of CMVs to disease progression (in particular, inflammation and cancer) has been well established [108,110]. Interestingly, data from zebrafish and mouse models have shown that exosomes isolated from brain endothelial cells have the ability to overcome the BBB and deliver their content to CNS despite their size around 30–100 nm. Thus, exosomes represent a new cutting-edge in the field of nanocarriers [108] and their application in VitB12, in our opinion, could represent a very promising treatment in the case of neuropathologies, albeit no data are currently available. 

## 7. Are Nano-Based Formulations of VitB12 Affordable?

The nanotechnologies applied to the VitB12 delivery, and more in general applied to drug delivery, is currently in an embryonic state. Scientists still questioned if nanocarriers can be safe. For example, liposomes are associated with different side effects [111,112] even if the developing of new nanomedicine patient-friendly drug delivery systems are rising [113]. A lot of knowledge gap is due to the undescribed processes regarding the absorption, distribution, metabolism, and excretion of nanocarriers, and very few treatments involving them have been approved so far [68]. 

The development of the therapy for the administration of VitB12 dates back to the 1960s. The first studies tried to establish the blood concentration of the vitamin in the plasma and in the organs after administration of it through oral prescription or intramuscular injection. The latter has been chosen as a medical treatment routine worldwide for several reasons as discussed before. In Canada and Sweden, the oral VitB12 option is available: in Sweden, the replacement reaches 73% of the total cases without observing inappropriateness [114], and in Canada, it has been estimated in 2001 that the switch to oral prescription could have saved up to $17.6 million over 5 years in Ontario [115]. More in detail, van Walraven and coworkers estimated that if all elderly patients receiving vitamin B12 (cobalamin) injections in 2001 were switched to high-dose oral therapy, between $2.9 million and $17.6 million would be saved [116]. On the other hand, we must consider the cost of producing nanoparticles. To make an in-depth analysis for each nanoparticles/nanocarrier is quite difficult, but, for example, if we consider the expensive first-generation PEGylated liposomal doxorubicin with gemcitabine, the cost-effectiveness analysis reveals that its use leads to a cost saving [117]. Furthermore, we should also consider that the improvement of technologies will lead to a decrease in the cost of producing while the health care people needed to the injection (and all side costs such as commuting of the patient to the hospital) will not change in the next future. Furthermore, few studies investigate in-depth the fate of the VitB12 after administration such as stability, pharmacokinetics, and excretion while the safety is established. Indeed, cyanocobalamin is considered very safe, and since the 1970s, about 1700 cases have been reported with mild adverse effects, while the results of the hypercobalaminemia are still not well established [118].

## 8. Conclusions

The need of VitB12 supplementation is caused by several defects including alteration in absorption by the gastrointestinal tract, gastrointestinal surgery, dietary deficiency, defects in vitamin absorption or transport, and finally defects in vitamin metabolism. The limitations that could be associated with gastrointestinal absorption (although not determined) pushed researchers to consider other routes of treatment such as administration through the oral–buccal mucosa accessing thus developing very promising options. Studies that tested the efficacy and safety of these new prescriptions allowed us to broaden our knowledge on the bioavailability of the VitB12, for example. In this context, nanotechnologies, and in particular the use of nanocarriers, could help by improving both bioavailability and decreasing the frequency of dosing and making treatment easier for the patient and save money. Being very innovative, the safety of nanocarriers have not yet been well established and their use is limited. The papers reviewed here establish that the use of nanotechnologies definitely improves the effectiveness of VitB12 and suggest that the drug delivery can be engineered for precise targeting thus representing promising technology for patient-tailored therapy.
